# Supplementary material for: Radiomics-Based Predictive Model of Radiation-Induced Liver Disease in Hepatocellular Carcinoma Patients Receiving Stereo-Tactic Body Radiotherapy
Source: Biomedicines. 2022 Mar 3;10(3):597. doi: 10.3390/biomedicines10030597 (PMC8945465; doi:10.3390/biomedicines10030597)
Supplement: Supplementary file 1 [file biomedicines-10-00597-s001.zip › biomedicines-1578756-supplementary.pdf]

Comparisons of Model Validation Between Hold-out and Cross Validation Approaches

For the RF model, the F1 score was 0.980 ± 0.060 and 0.857; the accuracy was 0.989 ± 0.033 and 0.944; the AUROC was 0.993 ± 0.021 and 0.956 in the cross validation and the original hold-out method, respectively. And for the LR model, the F1 score was 0.760 ± 0.184 and 0.8; the accuracy was 0.894 ± 0.083 and 0.944; the AUROC was 0.918 ± 0.090 and 0.889 in the cross validation and the original hold-out method, respectively. The detail results of the comparison of model performance between 10-fold cross validation and the original proposed hold-out method was summarized in the Table S1. Based on the cross-validation data, we found a high sensitivity and positive predictive rate with small standard deviations of the proposed models.

Table S1: Model performance for prediction of RILD using random forest and logistic regression model for hepatocellular carcinoma treated with SBRT

	Random Forest (10-fold CV)	Random Forest (hold out 20%)	Logistic Regression (10-fold CV)	Logistic Regression (hold out 20%)
Sensitivity	1.000 ± 0.000	1.000	0.950 ± 0.150	0.667
Specificity	0.986 ± 0.042	0.933	0.888 ± 0.090	1.000
Positive predictive rate	0.967 ± 0.100	0.750	0.667 ± 0.236	1.000
Accuracy	0.989 ± 0.033	0.944	0.894 ± 0.083	0.944
F1 score	0.980 ± 0.060	0.857	0.760 ± 0.184	0.800
AUROC	0.993 ± 0.021	0.956	0.918 ± 0.090	0.889

*SBRT:stereotactic body radiation therapy, RILD: radiation-induced liver disease, CV: cross validation, AUROC: area under receiver operating characteristic

Table S2 Representative cases for the predictions of RILD (Test Case #15 and #16)

Patient	Test Case #15	Test Case #16
Sex	Male	Male
Age	82	39
ALBI grade	2	2
Child pugh score	A5	A5
Prescribed dose	50 Gy / 5 fractions	50 Gy / 5 fractions
Normal liver volume (cc)	960.12	2315.53
GTV volume (cc)	62.33	179.78
V5 (%)	69.3	73.22
V15 (%)	25.32	24.63
V30 (%)	8.34	8.28
Difference Average	0.511	0.487
Strength	0.056	0.066
RILD	Neg	Pos
Predict with radiomics		
LR (0.531)[a]	Neg (0.476)[b]	Pos (0.653)[b]
RF (0.456)[a]	Neg (0.278)[b]	Pos (0.910)[b]

Predict without radiomics		
LR (0.331)[a]	Neg (0.040)[b]	Neg[c] (0.080)[b]
RF (0.129)[a]	Pos[d] (0.502)[b]	Pos (0.622)[b]

*RILD: radiation-induced liver disease, ALBI: albumin-bilirubin, cc: Cubic Centimeter, GTV: gross tumor volume, LR: logistic regression, RF: random forest, Neg: negative, Pos:positive

[a] *best cut-off value* [b] *possibility score calculated from predictive model* [c] *false negative* [d] *false positive*

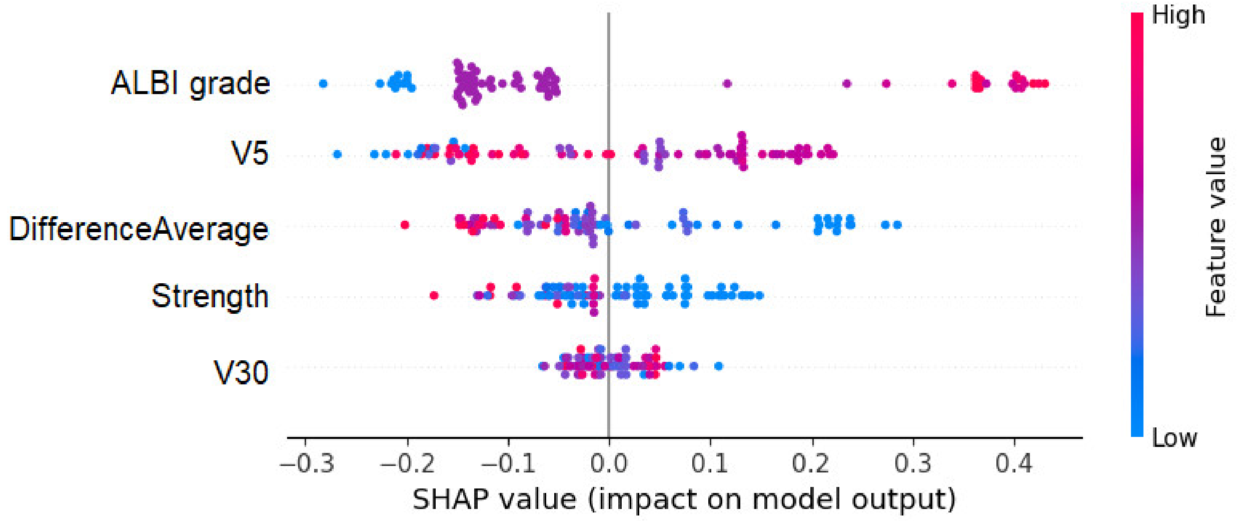

Figure S1. Color labeling of each sample point indicates the feature value (red for the high values and blue for low values). The horizontal axis represents the SHAP value reflecting the imapct of feature on model decision. Combining the feature values (color of points) and its distribution along the horizontal axis, we could observe the association of feature value with the possibility of RILD occurrence.
